# A Comparative Analysis of Enamel Surface Roughness Following Various Interproximal Reduction Techniques: An Examination Using Scanning Electron Microscopy and Atomic Force Microscopy

**DOI:** 10.3390/biomedicines12081629

**Published:** 2024-07-23

**Authors:** Dan-Cosmin Serbanoiu, Aurel-Claudiu Vartolomei, Dana-Valentina Ghiga, Marioara Moldovan, Codruta Sarosi, Ioan Petean, Marie-Jose Boileau, Mariana Pacurar

**Affiliations:** 1 Faculty of Dental Medicine, GEP University of Medicine Pharmacy, Science and Technology of Targu Mures, 540139 Târgu Mures, Romania; serbanoiu.dancosmin@gmail.com (D.-C.S.); claudiu.vartolomei@gmail.com (A.-C.V.); valentinaghiga@gmail.com (D.-V.G.); marianapac@yahoo.com (M.P.); 2 Raluca Ripan Chemistry Research Institute, Babes-Bolyai University, 400294 Cluj-Napoca, Romania; codruta.sarosi@ubbcluj.ro; 3 Faculty of Chemistry and Chemical Engineering, Babes-Bolyai University, 400084 Cluj-Napoca, Romania; ioan.petean@ubbcluj.ro; 4 Faculty of Dental Medicine, Bordeaux University, 33076 CEDEX Bordeaux, France; mariejoseboileau@gmail.com

**Keywords:** atomic force microscopy, scanning electron microscopy, enamel surface roughness, interproximal reduction

## Abstract

Interproximal enamel reduction (IER) is a minimally invasive therapeutic procedure commonly used in orthodontics to address both functional and aesthetic issues. Its mechanical effects on enamel surfaces induce the formation of grooves, furrows, scratches, depressions, and valleys. The aim of this study was to assess the enamel surface roughness resulting after the application of currently available methods for interproximal reduction. Ninety freshly extracted human teeth were divided into six groups and subjected to the stripping procedure, using a different method for each group (diamond burs, abrasive strips of 90 μm, 60 μm, 40 μm, 15 μm, and abrasive discs). A single individual performed stripping according to the manufacturer’s recommendations, involving interproximal reduction on one tooth’s proximal face and leaving the other side untreated. Qualitative and quantitative assessment of the enamel surfaces was carried out using Scanning Electron Microscopy (SEM) and Atomic Force Microscopy (AFM), obtaining 2D and volumetric 3D images of the enamel surface microstructure and nanostructure. The study found that diamond burs and abrasive strips of 60 μm and 90 μm increased enamel roughness due to intense de-structuring effects, while the 40 μm polisher had a gentler effect and 15 μm abrasive strips and polishing discs preserved enamel surface quality and removed natural wear traces.

## 1. Introduction

Interproximal enamel reduction (IER), alternatively referred in the literature to as enamel reduction, interdental stripping, air rotor stripping, slenderizing or reproximation, encompasses the process of selectively removing enamel from the mesial or distal surfaces of the teeth [1]. The technique is indicated in the majority of cases in adults primarily due to dental morphology and pulp position, and secondarily due to patient motivation—particularly aesthetic concerns—as well as the necessity of a rapid treatment without dental extractions [2]. Moreover, enamel is considered structurally immature only for the first 2–4 years after eruption into the oral cavity [3]. This maturity is critical as it affects the outcomes of dental procedures like inter-proximal reduction (IPR).

Most often, this is utilized in dento-maxillary disharmonies with crowding as a method of creating space in the dental arches with minimal invasiveness or to correct Bolton discrepancies both in fixed and removable orthodontic therapy, truly gaining momentum with the advent of orthodontic therapy with aligners [4,5,6,7]. It can be utilized both in the anterior and posterior dental segments; however, it requires, prior to its implementation, a critical and careful analysis of the dental morphology and the level of oral hygiene of the patient. These two conditions are imperatively necessary and must be respected for the practice of the stripping therapeutic procedure [5,8]. Through interproximal enamel reduction and coronal recontouring, in addition to space creation, interdental black triangles are also eliminated—thereby gaining an additional harmony through the presence of more stable contact points and greatly improved smile aesthetics in the adult patient [9].

Dental enamel, an epithelial-derived mineralized structure, constitutes a protective covering at the level of the tooth crown and is renowned as the hardest tissue in the human body, comprised of 92–96% inorganic matter or mineral phase, 3% water, and 1% organic material by weight [10,11].

The mineral structure of dental enamel is composed of hydroxyapatite (HAP) molecules, Ca_10_(PO_4_)_6_(OH)_2_, which exhibit a complex hierarchical assembly spanning from the nanometric to the microscopic scale and which possess anisotropic properties [12,13,14].

The overall microstructural appearance of dental enamel contains a dense and orderly network of such hydroxyapatite prisms with a honeycomb-filled-with-honey appearance. In the case of acidic erosion, this attacks the core of the HAP prisms, causing a loss of mineral material so that the appearance becomes like an empty honeycomb. This aspect has been highlighted in the scientific literature through Scanning Electron Microscopy (SEM) and Atomic Force Microscopy (AFM) [15,16].

The surface appearance of dental enamel is not smooth; it exhibits a series of irregularities in the form of peaks, ridges, depressions and valleys, originating from its onto-phylogenetic development [17]. Thus, the enamel surface can be assessed through a set of parameters—surface roughness—which provide information regarding the height and distribution of surface elements [18,19].

Enamel surface roughness can be assessed using various methods including profilometry, rugosimetry, SEM, and AFM. Profilometry, rugosimetry, and AFM yield three-dimensional (3D) numerical data on surface roughness, facilitating subsequent evaluation. Conversely, SEM provides two-dimensional (2D) information, necessitating the use of visual enamel evaluation indices for statistical analysis [20].

Interproximal reduction (IPR) unavoidably modifies the tooth enamel, leading to alterations in both the enamel surface morphology and contour. Various qualitative studies have indicated that the removal of this outer enamel layer results in the formation of numerous grooves and furrows on the tooth surfaces [1,21,22,23].

Ensuring a comprehensive finish of the enamel surfaces after treatment is essential for a favorable long-term outlook of the stripped teeth, as any remaining roughness can contribute to plaque accumulation, leading to demineralization or the formation of carious lesions. Therefore, it is imperative to undertake proper post-processing with suitable equipment [24].

Using SEM, grooved and roughened enamel surfaces have been observed on the interproximal enamel of both deciduous and permanent teeth [1]. Furthermore, deciduous enamel exhibits smoother surfaces compared to permanent enamel [18]. However, SEM studies provide only a subjective assessment of surface roughness. There are few quantitative studies of enamel following IPR, primarily focusing on surface roughness (Ra) [22,24].

It has been noted that stripping leads to an increase in surface roughness, regardless of the instruments utilized. This increased roughness may elevate the susceptibility of stripped enamel to bacterial adhesion and biofilm formation, which could then be shielded from mechanical clearance by salivary flow, brushing, or flossing—potentially promoting demineralization and the accumulation of plaque and calculus [25].

Previous studies have demonstrated that the initial microbial colonization of the enamel surface begins in surface irregularities such as grooves, perikymata, cracks, and abrasion defects, and subsequently spreads from these areas. In vivo data have shown that rough surfaces significantly facilitate plaque retention and can harbor bacteria by up to 25 times more than their smooth counterparts [26,27]. Dental surfaces with high enamel roughness polarize the adherence of bacterial plaque, leading to a drastic decrease in pH, chemical dissolution of the enamel, and the occurrence of dental caries. A reduction in roughness results in a remarkable decrease in plaque formation [28]. Therefore, efforts to achieve the lowest possible enamel roughness should be considered when performing therapeutic stripping operations. A surface roughness value of the enamel of below 200 nm is necessary to prevent bacterial adhesion and plaque accumulation [29].

AFM represents a valuable tool for examining teeth, offering not only visual images but also quantitative data on parameters like roughness, periodicity, and mechanical properties [30,31,32]. It holds significance in identifying the inherent surface characteristics of teeth, particularly when investigating potential interactions and modifications induced by materials and treatments [12].

The objectives of this study were to examine the micro and nanotopography of enamel surfaces generated by commonly employed IPR instruments and to assess the impact of IPR procedure on these surfaces. The null hypothesis tested was that there is no relationship between enamel surface roughness and the various methods of interproximal reduction.

## 2. Materials and Methods

This study was conducted in accordance with the Declaration of Helsinki, and approved by the Ethics Committee of George Emil Palade University of Medicine, Pharmacy, Science and Technology of Targu Mures, Romania (No. 2157 from 20 March 2023).

Ninety human permanent teeth, extracted from young adult patients treated at the Faculty of Dental Medicine of George Emil Palade University of Medicine, Pharmacy, Science and Technology in Târgu Mureș for orthodontic purposes or as a result of severe periodontal damage were collected using the following exclusion criteria: staining, demineralization, severe decay, fluorosis, cracks or defects in the enamel, and prosthetic restorations on the mesial or distal tooth surfaces. 

The inclusion criteria for the crowns required a normal morphology, intact enamel, and no history of orthodontic treatment. The 90 teeth selected for this study were obtained from young adult patients aged between 18 and 30 years to minimize variability in enamel quality.

The teeth were thoroughly washed in running water and all blood, adherent soft tissues, and calculus were removed through gentle mechanical cleaning with a toothbrush of medium hardness.

After cleaning, the teeth were disinfected by placing them in a 1.0% chloramine-T trihydrate bacteriostatic/bactericidal solution (pH = 9.1) for a maximum of one week in accordance with the specifications of ISO 3696:1987 (International Organization for Standardization ISO/TS 11405, Third Edition, 1 February 2015) [33]. Subsequently, to simulate conditions in the oral cavity, all teeth were stored in an artificial saliva solution. To minimize deterioration, the storage medium was replaced at least once every two months. The artificial saliva was comprised of disodium phosphate, sodium bicarbonate, calcium chloride, hydrochloric acid, and water [34], and it was prepared at the Raluca Ripan Institute for Research in Chemistry in Cluj Napoca, adhering to findings identified in other research studies [35]. The percentages are presented in Table 1.

To simulate in situ conditions, the teeth were embedded into a fixed plaster model. In order to replicate a degree of natural tooth movement within the plaster model, silicone material (Optosil; Heraeus Kulzer, Hanau, Germany) was used to mount the teeth instead of merely embedding them directly into the plaster [24]. All enamel stripping was carried out by the same operator and according to the manufacturers’ instructions for each instrument. For the burs, the hand pieces were run at 400,000 rpm with water cooling; for the discs and the abrasive strips, the hand pieces were run at 5000 rpm [1]. 

The stripping instruments used were represented by 8392 “mosquito” bur fine grit stripping burs (red color code), size 0.16, manufactured by Komet USA. Regarding the stripping strips, all four sizes were used: 15 microns, 40 microns, 60 microns, and 90 microns, operated with a handpiece for stripping manufactured by Task Inc at a speed of 5000 rpm and with oscillatory movements of 1.3 mm. The abrasive discs used for stripping were Sof Lex polishing discs (fine), manufactured by 3M Germany, with an orange color code—abrasive.

Each IPR instrument (i.e., bur, strip, and disc) was used for 1 enamel sample only and then replaced. To ensure an equal reduction of enamel across all teeth, the IPR was carried out over the entire thickness of the stripping instrument (maximum 0.5 mm), which was activated for 30 s on the enamel surface [36]. This duration was also chosen based on standard dental practices. The 90 teeth were divided into 6 groups corresponding to each stripping instrument used, as shown in Table 2. Thus, each group of teeth included 15 entities, with the therapeutic stripping procedure performed on either the mesial or distal side of the tooth, while the enamel on the opposite side was left untouched as a control.

Following the completion of IPR, the samples were cleaned with a 0.9% saline solution and dried using the air spray from the dental unit. Subsequently, they were placed and stored in artificial saliva until the SEM and AFM measurements were conducted.

Samples of mechanically processed teeth were presented for investigation, affected by the action of various stripping devices as follows: burs; abrasive particle polishers with diameters of 90, 60, 40, or 15 μm; and polishing discs. The procedure involved slicing thin enamel sections from the processed area for SEM and AFM investigations, as well as a slice from the unprocessed part as a control.

Scanning Electron Microscopy was conducted using an Inspect S microscope produced by the FEI Company (Hillsboro, OR, USA). The samples were examined in low-vacuum mode at an accelerating voltage of 25 kV.

Following the SEM investigation, the tooth samples were sectioned using the IsoMet 100 microtome, manufactured by Buehler, Coventry, UK, to meet the requirements of the AFM investigation. The slices were cut parallel to the enamel surface to ensure optimal positioning on the AFM sample holder.

Atomic Force Microscopy was performed using a JEOL JSPM 4210 microscope (Tokyo, Japan), utilizing the intermittent contact mode. The probe used to palpate the sample was a type NSC 15 Hard produced by MikroMasch (Sofia, Bulgaria), with a resonance frequency of 325 kHz and a force constant of 40 N/m. The fine microstructure of the dental enamel was investigated over a scanning area of 5 μm × 5 μm, and its nanostructure over an area of 1 μm × 1 μm. The images were analyzed using the specialized software Win SPM 2.0 produced by JEOL (Tokyo, Japan), measuring the surface roughness values of the scanned area by following the linear Ra and Rq parameters. Ra represents the arithmetic average of the profile height and is described by Equation (1), and Rq represents the root mean square of the profile height and is described by Equation (2):

(1) $$Ra=\frac{1}{l_{r}}\int_{0}^{l_{r}}\left|z(x)\right|dx$$

and

(2) $$Rq=\sqrt{\frac{1}{l_{r}}\int_{0}^{l_{r}}\left|z{\left(x\right)}^{2}\right|dx}$$

where l is the total length of the profile and z is the height measured at point x [37].

All enamel slices from each group were evaluated and imaged in 3 randomly selected areas (10 µm × 10 µm, 5 µm × 5 µm, 2.5 µm × 2.5 µm, 2 µm × 2 µm, and 1 µm × 1 µm AFM areas); 2D topographic images and surface plots were made to obtain a three-dimensional perspective of the surface, from which the average surface roughness values for the peak height, Ra, and Rq were calculated.

The statistical analysis encompassed elements of descriptive statistics (mean, median, standard deviation) and inferential statistics. The Shapiro–Wilk test was employed to determine the distribution of the analyzed data series. For comparing more than two data sets with non-Gaussian distribution, a nonparametric Kruskal–Wallis test was used, and for multiple comparisons, Dunn’s test was applied.

Student’s *t*-test, a parametric test for comparing means, was applied, as well as the Mann–Whitney U test—a non-parametric test for comparing medians. The significance threshold chosen for the *p*-value was 0.05. The statistical analysis was conducted using trial version 10 of the GraphPad Prism software, Dotmatics, Boston, MA, USA.

## 3. Results

### 3.1. Scanning Electron Microscopy (SEM)

**Figure 1:**
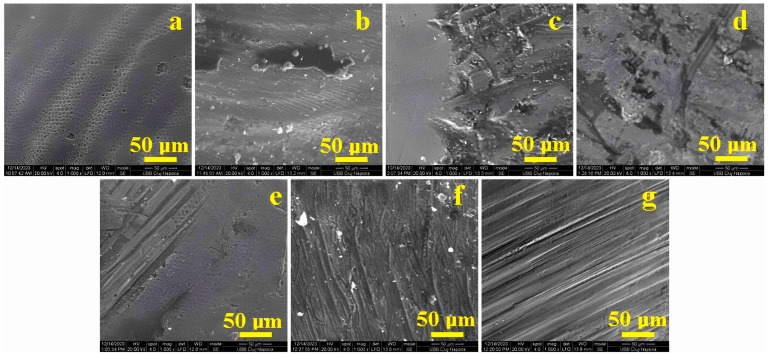
SEM images of the overall microstructure of the polished enamel surface using different devices: (**a**) control sample—unpolished enamel, (**b**) burs, (**c**) 90 µm, (**d**) 60 µm, (**e**) 40 µm, (**f**) 15 µm, and (**g**) discs.

Considering fundamental natural aspects related to the dental enamel surface, an investigation into the overall microstructure of the samples was undertaken using SEM at a magnification of 1000×, as shown in Figure 1. The control sample—Figure 1a—exhibited the microstructural appearance of a healthy tooth with normal wear marks due to mastication, but without lesions or defects that could be correlated with certain conditions. Thus, the enamel surface appeared wavy due to prolonged contact with the translational movement of masticated food relative to the tooth, showing a periodicity with gentle rises of a thickness of about 50 µm and lengths exceeding the image frame (thus, over 500 µm). The peak of these elevations was quite blunt in the direction of food pressing, and the opposite side showed slight signs of acidic demineralization, with the honeycomb-like appearance being visible—the observed depressions being about 5 µm in diameter.

Grinding the enamel surface destroyed its natural appearance, inducing severe abrasion marks in the form of circular grooves parallel to the major radius (depending on the geometry of the bur) with a periodicity of about 70 µm, as shown in Figure 1b. Over these abrasion grooves, islands of debris appeared with an irregular elongated shape in the direction of the abrasion, which had a length of 150 µm and a width of 40–45 µm.

The enamel surface prepared with 90 µm abrasive strips exhibited a profoundly altered overall microstructure due to the overlay of abrasion channels in well-concentrated areas such as the central and right parts of the observation field of Figure 1c, while the left side displayed a smooth enamel area unaffected by the abrasive material of the strips.

The enamel surfaces treated with 60 µm abrasive strips—Figure 1d—and, respectively, with 40 µm strips—Figure 1e—exhibited abrasion marks in the form of parallel striation bands induced by the abrasive material, the width of which corresponded to the diameter of the abrasive material. This led to a relatively irregular surface that presented with treatment-induced depressions in places.

Strips with 15 µm abrasive material led to an overall microstructure with a succession of parallel striation bands of approximately 15 µm, grouped in wide strips of 45–55 µm, as shown in Figure 1f. This resulted in a uniformization of the enamel surface by eliminating the wavy appearance of the untreated portions.

Ultimately, the sample treated with polishing discs presented a uniform appearance with parallel striations induced by the mechanical treatment, whose width is about 1 µm, but many of these fell into the submicron range, necessitating further investigation.

### 3.2. Atomic Force Microscopy (AFM)

**Figure 2:**
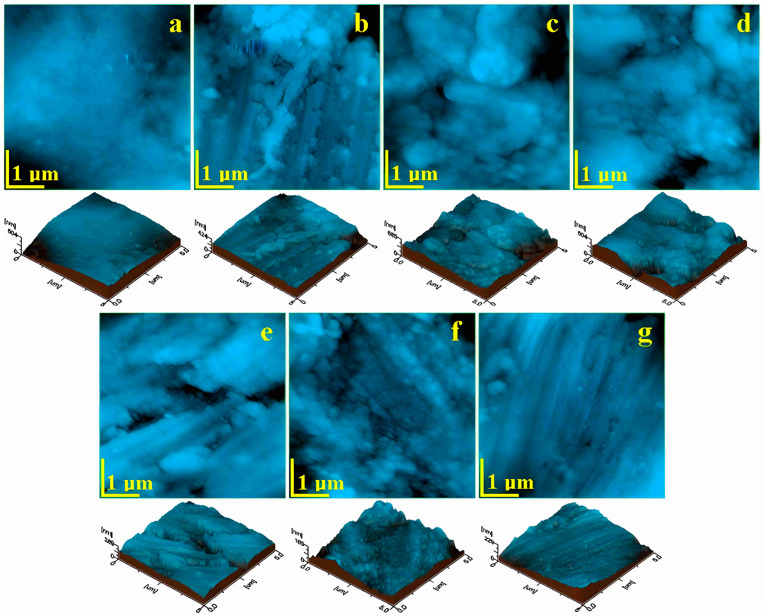
AFM images of the fine microstructure of the enamel surface treated with various devices: (**a**) control sample—unpolished enamel, (**b**) burs, (**c**) 90 µm, (**d**) 60 µm, (**e**) 40 µm, (**f**) 15 µm, and (**g**) discs. The three-dimensional profile is presented beneath each topographic image individually.

**Figure 3:**
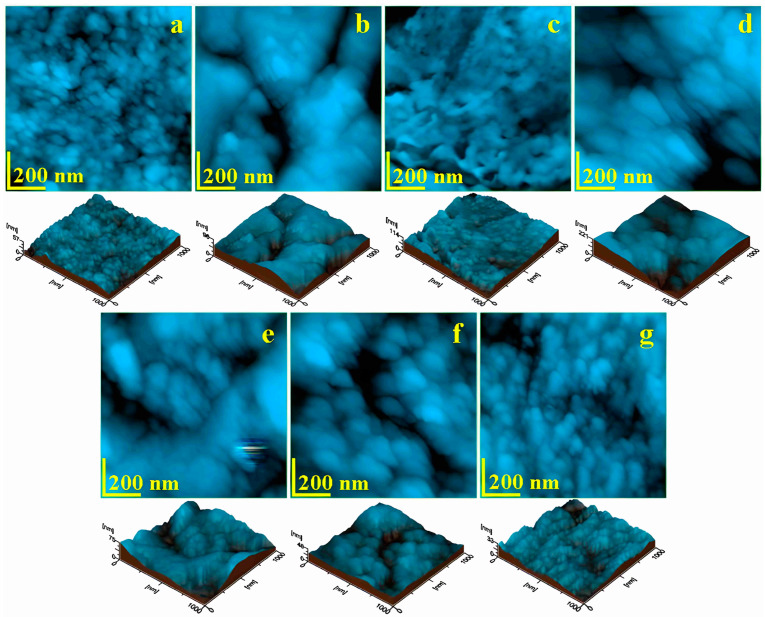
AFM images of the nanostructure of the enamel surface treated with various devices: (**a**) control sample—unpolished enamel, (**b**) burs, (**c**) 90 µm, (**d**) 60 µm, (**e**) 40 µm, (**f**) 15 µm, and (**g**) discs. The three-dimensional profile is presented beneath each topographic image individually.

Using the Atomic Force Microscope, the microstructural and nanostructural details of the prepared enamel surfaces were studied at a scanning area of 5 µm × 5 µm to capture the appearance of the hydroxyapatite prisms, as well as the fine microstructure of the enamel—as shown in Figure 2—and the enamel nanostructure at a scanning area of 1 µm × 1 µm, Figure 3.

**Figure 4:**
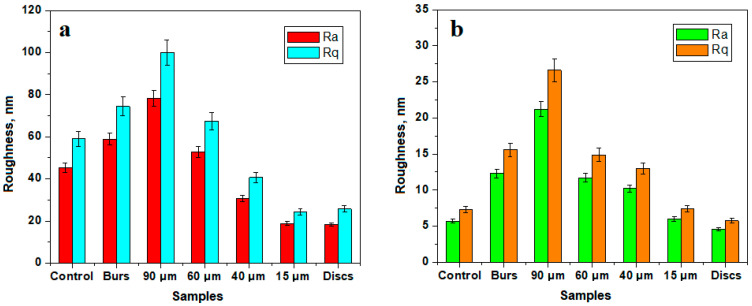
Variation of surface roughness parameters Ra and Rq for: (**a**) fine microstructure and (**b**) nanostructure.

Thus, the control sample (unprocessed), Figure 2a, displayed a smooth surface with very compact nanostructural units but also exhibited a wavy appearance, which is in good agreement with the SEM observation. The upper right and lower left corners showed lower portions that correlated well with the slightly acid-eroded areas of two adjacent HAP prisms. This resulted in relatively high values of surface roughness—Figure 4a.

After grinding, the fine microstructure of the enamel presente a very uneven topography—Figure 2b—which highlighted at the top of the scanned area an agglomeration of abrasion debris, and in the middle and bottom parts, a succession of parallel striations induced by abrasion was observed. Although grinding completely removed the wavy structure of the enamel, it was replaced with unevenness induced by the bur that resulted in an increased value of roughness, as shown in Figure 4a.

The 90 µm abrasive strips induced a very rugged topography due to the intersection of abrasion channels; thus, we observed roughness portions with relatively rounded edges with diameters of between 1 and 3 µm, bordered by abrasion striations alternated with some depressions of about 1.5 µm in diameter, such as the one in the upper right corner of Figure 2c. These unevennesses at the level of the fine microstructure of the enamel led to the highest roughness value observed, as shown in Figure 4a.

The strip with 60 µm abrasive material led to a fine microstructure that was somewhat similar, having bouldery structures with diameters of 1–3 µm, but harmoniously blended together without abrasion grooves; however, some depressions still appeared, such as the one in the lower middle part of Figure 2d. This resulted in a slightly lower surface roughness value compared to that resulting from the 90 µm strips.

Figure 2e captures the fine microstructural details that occurred between the abrasion striations induced by the 40 µm abrasive strips. Although the overall topography of the enamel was quite smoothed compared to the initial undulations, there were quite deep grooves with a width of about 200 nm, a length of over 4 µm, and a depth of about 50 nm, as observed from the three-dimensional profile presented beneath the AFM image. This resulted in significant roughness values, but they were considerably smaller compared to those obtained by the 60 µm abrasive strips—Figure 4a.

The 15 µm abrasive strip produced a quite-uniform, fine microstructure of the dental enamel, as shown in Figure 2f—completely removing the initial wavy aspect and ensuring a compactness and smoothness of the surface that led to a low surface roughness value. However, the abrasion striations were still perceptible at the level of the fine microstructure through the low areas in the upper right and lower left corners of Figure 2f. Through the action of the polishing abrasive discs, these fine abrasion striations were eliminated, making the surface smoother and more uniform—Figure 2g. The polishing effect proved to be more cosmetic, since the roughness value was almost identical to that obtained with the 15 µm abrasive strip—Figure 4a.

The nanostructural level is very important for healthy enamel and is characterized by hydroxyapatite nanostructural units with diameters ranging between 40 and 60 nm that are well-fused together into a smooth and compact surface, as seen in the standard sample of Figure 3a. The roughness of this surface was about 5 nm for Ra and 7 nm for Rq, as observed in Figure 4b.

Topographic details at the structural level indicated a profound disorganization of the dental enamel as a consequence of the milling process. The uniform and compact structure became rugged, featuring compact insular domains with a dendritic appearance separated by deep scratches—Figure 3b. This was due to the combined effect of milling scratches and the submicronic clustering of debris, resulting in a significant increase in roughness at the nanostructural level, as also shown in Figure 4b.

Another category of advanced destruction of the dental enamel nanostructure was induced by mechanical treatment with 90 μm abrasive strips, which literally scraped away large portions of the nanostructure. Thus, the nanostructural units lost their consistency and disintegrated under the effects of the tangential stresses induced by the shock generated by the moving abrasive particles—Figure 3c. The immense energy dissipated at the nanostructural level led to the breaking of the HAP units and their advanced fragmentation, such that their boundaries were no longer visible, resulting in a kneaded mass with elevations and valleys—indicating the loss of cohesion provided by the protein binder. Consequently, the roughness reached a maximum value as a result of the mechanical treatment with 90 μm abrasive strips, as observed in Figure 4b.

The 60 μm abrasive strip also had a drastic effect on the nanostructure of the enamel, Figure 3d. The nanostructural units were subjected to increased stress, causing them to deform—being relatively elongated in the direction of abrasion—and their average diameter was around 120 nm. This indicates a weakening of their internal cohesion through the partial destructuring of the protein bonds. The fact that the HAP nanostructural units were not completely disorganized ensures a certain nanostructural cohesion, reflected by a lower value of roughness—Figure 4b. Generally, the same effect of the relative destructuring of the nanostructure was induced by the treatment with 40 μm strips, but the nanostructural units were not as affected—Figure 3e. Therefore, the surface roughness was significantly reduced compared to the value resulting from the 60 μm strips—Figure 4b.

Mechanical treatment with 15 μm abrasive strips had a gentler effect on the enamel nanostructure, causing only a slight increase in the diameter of the nanostructural units to about 70 nm—Figure 3f—but ensuring good surface cohesion. This led to roughness values comparable to those of the standard nanostructure, as also shown in Figure 4b.

Finally, treatment with polishing discs ensured a uniformization of the nanostructure of the processed enamel, smoothing local roughness and preserving the HAP nanostructural units, which maintained their diameter of 40–60 nm, as evident from Figure 3g. This led to surface roughness values slightly lower than those of the control sample—Figure 4b.

Analyzing the variation of the enamel surface roughness values at both the microstructure and nanostructure levels, Figure 4, we can observe that burs and abrasive strips of 60 and 90 μm caused a significant increase in enamel roughness due to the intense destructuring effects induced. On the other hand, the 40 μm abrasive strips had a gentler effect on the dental enamel, while the 15 μm strips and polishing discs ensured good preservation of the enamel surface quality and also ensured the removal of natural wear traces present in the control sample. For the quantitative analysis of enamel surfaces after IPR, the various stripping instruments used produced different degrees of enamel surface roughness. Generally, in the statistical analysis of parameters such as Peak Height, Ra, and Rq, the 90 μm abrasive strips produced the roughest surfaces, followed by diamond burs, compared to the control enamel samples (*p* < 0.05).

Regarding the Peak Height parameter, the highest values were recorded after using the 90 μm abrasive strips, achieving 602.2 ± 501.5 nm, followed by the use of diamond burs for stripping, which reached 540 ± 681 nm (Table 3). 

As can be observed in Table 3, there was a statistically significant difference between the medians of the Peak Height (nm) values in the analyzed groups (*p* < 0.05).

**Figure 5:**
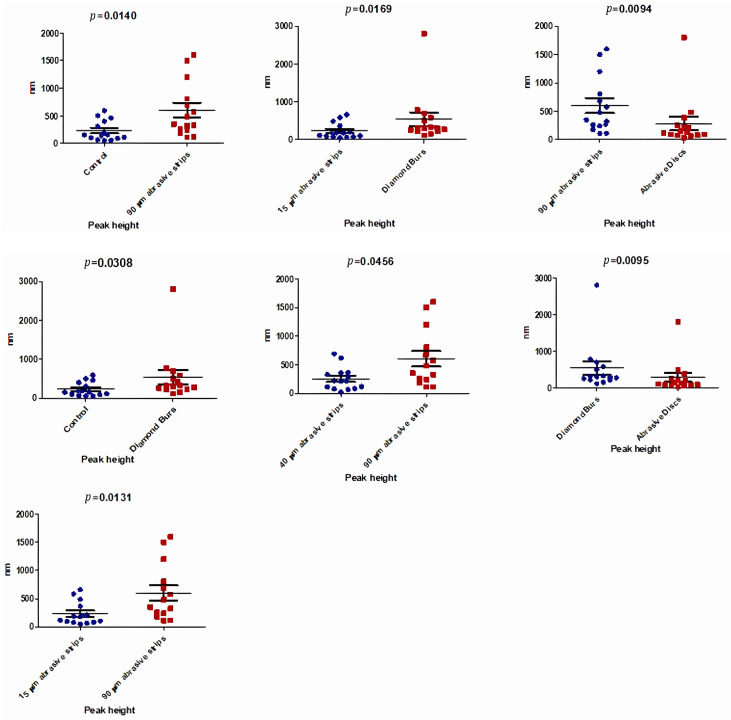
Comparative analysis between sample groups (Peak Height Parameter).

Statistically significant differences were identified in the comparisons between the values measured after the use of diamond burs and the control group (*p* = 0.0308), and between the 90 μm abrasive strips and the control group (*p* = 0.0140), as shown in Figure 5.

The lowest value of the Peak Height parameter was recorded after the use of 15 μm abrasive strips (232.5 ± 206.9 nm), comparable to that of the enamel in the control group (231.1 ± 185.8 nm). Additionally, reduced values were also obtained after using 40 μm abrasive strips and polishing discs: 254.1 ± 204.8 nm and 284.5 ± 455 nm, respectively (Table 3). Statistically significant differences were observed between the Peak Height values obtained using 15 μm abrasive strips and the Peak Height values obtained through enamel grinding with diamond burs for stripping, as shown in Figure 5.

In Table 4, it can be observed that there was a statistically significant difference between the medians of the Ra (nm) values in the analyzed groups (*p* < 0.05).

**Figure 6:**
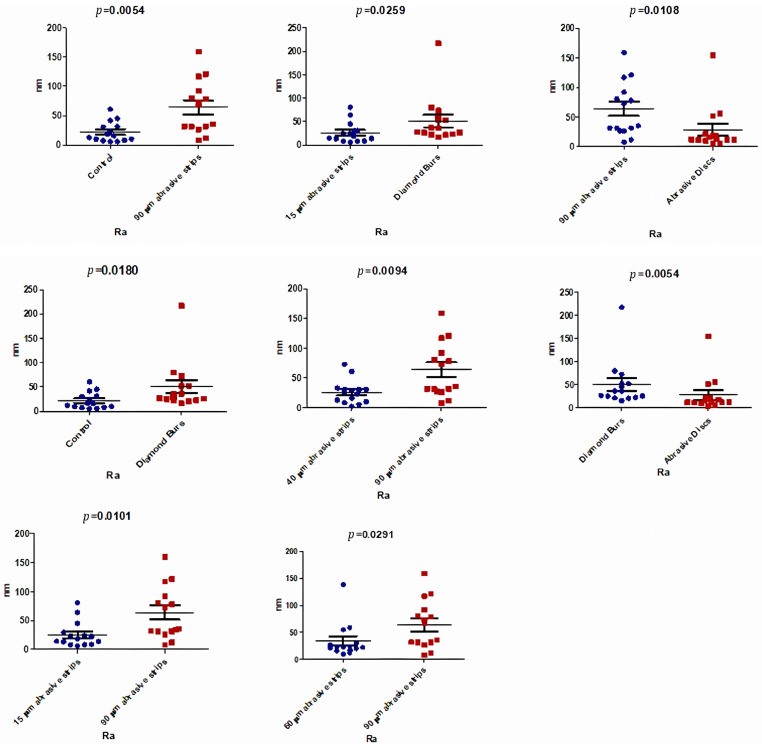
Comparative analysis between sample groups (Ra Parameter).

Thus, regarding the Ra parameter, similar values were obtained. The roughest surface was achieved after using the 90 μm abrasive strips (Ra, 64.04 ± 46.15 nm), which was significantly higher compared to the value obtained for the control group (Ra, 22.01 ± 17.49 nm), as indicated in Table 4. Statistically significant differences were observed in the comparisons between the Ra values after using diamond burs and the control group (*p* = 0.0180), and between the 90 μm abrasive strips and the control group (*p* = 0.0054; Figure 6). 

Once again, the lowest values of the Ra parameter were obtained after using the 15 and 40 μm abrasive strips, thus ensuring the uniformity of the nanostructure of the processed enamel by smoothing local roughness and preserving the hydroxyapatite nanostructural units, as detailed in Table 4.

Statistically significant differences in the Ra parameter between the various analyzed groups are detailed in Figure 6.

**Figure 7:**
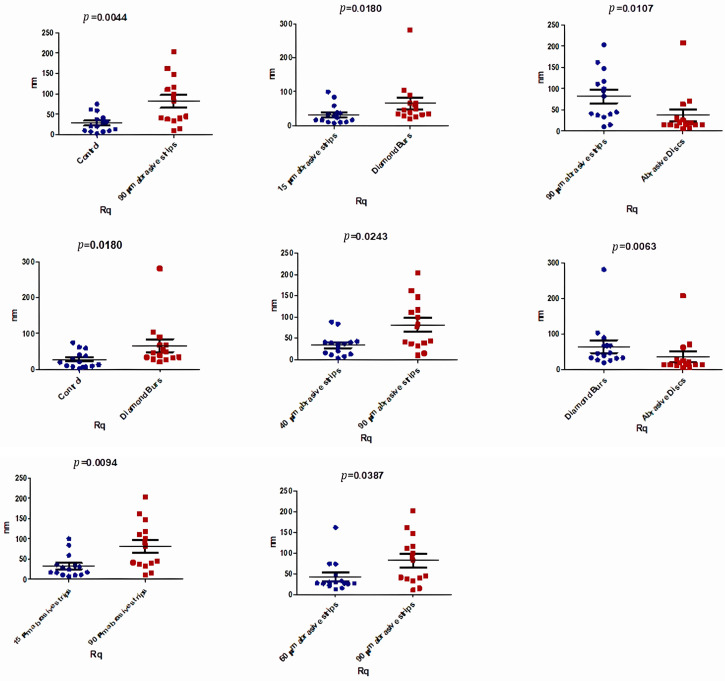
Comparative analysis between sample groups (Rq Parameter).

Regarding the Rq parameter, the roughest surface was created by the mechanical treatment with 90 μm abrasive strips (Rq, 81.69 ± 59.82 nm) and was significantly rougher (*p* = 0.0044) compared to the control group surface (Rq, 28.11 ± 23.37 nm). Additionally, statistically significant differences were also observed in the comparison between the Rq values after using diamond burs and the control group (*p* = 0.0180; Figure 7). Values close to those obtained for the control group were achieved after using 15 μm and 40 μm abrasive strips for stripping (Rq, 32.55 ± 28.82 nm) and (Rq, 34.29 ± 26 nm), respectively, as presented in Table 5.

Table 5 shows a clear and statistically significant difference in the medians of the Ra (nm) values in the analyzed groups (*p* < 0.05).

Statistically significant differences in the Rq parameter between the various analyzed groups are detailed in Figure 7.

Comparing the mechanical effects achieved by using different stripping instruments (i.e., burs, strips, and discs), the surfaces with the highest roughness were obtained following mechanical treatment with diamond burs and 90 μm abrasive strips, and were significantly rougher (*p* < 0.05) than the surfaces treated with 15 μm and 40 μm strips and polishing discs. These results, schematically presented in Figure 5, Figure 6 and Figure 7, hold true for all three roughness evaluation parameters discussed in the study (Peak Height, Ra, and Rq).

## 4. Discussion

Dental enamel is a highly hierarchical structure based on hydroxyapatite nanocrystals with dimensions of about 20 nm, which form nanostructural units of approximately 40–60 nm that are well-bonded together by protein material, forming prisms with a diameter of 5 µm. These hydroxyapatite prisms constitute the basic units of the fine microstructure of dental enamel [37,38,39]. 

Enamel surfaces can be categorized into prismatic and aprismatic types. Unlike prismatic enamel, where crystals are not aligned in a specific direction, aprismatic enamel features crystals that are parallel to each other [40]. This characteristic should be taken into account when utilizing native enamel for studies or treatments. Aprismatic enamel is predominantly found in the cervical, proximal, and occlusal areas, forming a layer with higher mineralization. Consequently, these regions should be avoided, as they lack a distinctive structural pattern [41,42,43].

In our study, the enamel nanostructure was highlighted using the Atomic Force Microscope (AFM). The enamel in control samples was characterized by HAP nanostructural units with diameters ranging between 40 and 60 nm, well-fused together into a smooth and compact surface. The roughness of the healthy enamel surface obtained in our study was approximately 5 nm for Ra and 7 nm for Rq, in full agreement with observations from the scientific literature on healthy enamel [44,45].

Today, dental extraction for orthodontic purposes has become increasingly less used, and as an alternative to obtain space on the dental arches, stripping has become more popular among orthodontists—especially in association with aligner orthodontic therapy [7,46]. 

While operative or restorative dentists exercise meticulous caution to preserve the integrity of the enamel on adjacent teeth during the preparation of an abutment or approximal cavity, orthodontic treatments deliberately involve the stripping or therapeutic reduction of enamel. During such procedures, it is imperative to proceed with heightened diligence due to the potential iatrogenic consequences, such as an elevated incidence of caries, periodontal disease, and heightened thermal sensitivity [7,47].

Numerous studies have demonstrated that interproximal enamel reduction is followed by an increase in the roughness of approximate enamel surfaces, resulting in greater retention of bacterial plaque and an elevated risk of dental caries as a consequence of residual furrows on the enamel surfaces subjected to IPR compared to untreated surfaces [4,21,48,49,50]. Yet, other research has not found a substantial link between enamel stripping and vulnerability to caries, indicating that enamel reduction does not necessarily make teeth more prone to caries. Furthermore, it is often observed that a period of demineralization is typically succeeded by natural remineralization of the hard dental tissue [51,52].

Previous studies have shown that high enamel surface roughness leads to increased adherence of bacterial plaque, which results in a decrease in pH, chemical dissolution of the enamel, and the promotion of dental caries. A reduction in roughness leads to a remarkable decrease in plaque formation [28]. Therefore, achieving the lowest possible enamel roughness should be considered during therapeutic stripping procedures. An enamel surface roughness of below 200 nm is necessary to prevent bacterial adhesion and plaque accumulation [29]. Since the highest enamel surface roughness range created in this study was in the group treated with 90 μm abrasive strips, with Ra values of 64.04 ± 46.15 (mean ± standard deviation) and Rq values of 81.69 ± 59.82 (mean ± standard deviation), the six instruments used for dental stripping appear to be clinically acceptable for performing orthodontic treatments safely.

For the quantitative assessment of enamel roughness induced through mechanical means, SEM proves to be of limited utility. Despite producing highly detailed images, scanning electron microscopy reveals information that is subjective and cannot be quantified [1]. In contrast to electron microscopy, atomic force microscopy facilitates a three-dimensional topographical analysis of specimens with minimal, if any, damage to the specimen surfaces. Our approach, which incorporates both atomic force and scanning electron microscopy along with the calculation of both Ra and Rq values, is expected to yield more reliable insights into the surfaces of enamel post-polishing compared with other studies [53,54]. Consequently, AFM was employed in our research to achieve both qualitative and quantitative evaluations of enamel surface roughness. 

The different techniques applied for interproximal reduction in this research exhibited notable variations in the level of surface roughness generated. The fine grit (red) diamond burs created the roughest surfaces, followed by 90 µm abrasive strips and 60 µm abrasive strips. This observation is consistent with earlier research findings that have highlighted the use of diamond-coated tools for increasing the roughness of enamel surfaces [23,24,47].

SEM and AFM imaging analyses have highlighted major alterations of the enamel surface following the grinding process and the action of the abrasive strips with 90 µm material, particularly through the destabilization of the binding protein material that ensures the cohesion between HAP nanoparticles. This fact is clearly evidenced by the deformation and increase in the diameter of the nanostructural units.

In this study, the surface roughness data indicated that all the tested stripping methods, except for the action of the Sof Lex abrasive discs, produced rougher surfaces than intact enamel. Multiple investigations have demonstrated that metallic strips induce profound grooves and abrasions that are amenable to elimination through polishing processes [21,23,55]. It has been articulated that the rougher the surface following interdental reduction, the more challenging it becomes to attain an impeccably smooth surface through subsequent polishing techniques [55].

In a study conducted by Arman et al., where scanning electron microscopy and profilometry were employed to assess the roughness of enamel surfaces after IPR, it was found that diamond-coated instruments created surfaces that were rougher than untreated enamel. This observation included the use of Sof-Lex discs for polishing after IPR. The surfaces subjected to polishing did not become smoother than the control surfaces, but resulted in surfaces that were smoother than the treated and unpolished surfaces following IPR [47].

Our research has shown that treatment with polishing discs ensures the smoothing of the enamel’s nanostructure by leveling local roughness and preserving the HAP nanostructural units, which maintain a diameter of 40–60 nm. This leads to surface roughness values slightly lower than those of the control sample. Thus, it was demonstrated that polishing can create an enamel surface smoother than all other surfaces, including untreated (control) surfaces in this study. This is in contrast to the study conducted by Arman et al., which reported that polishing with Sof-Lex discs reduced surface roughness, but did not reduce it to a level smoother than the control samples [47].

A possible initial explanation for the disagreement between these reports is that in our study, the Sof-Lex discs (abrasive, orange code, 20 μm) were used for 30 s, whereas the previous study employed only a single Sof-Lex disc (fine grit) for merely 20 s [47]. The use of a single disc for such a brief period might not have been sufficient to remove the roughness created by the IPR instrument. Furthermore, the most significant difference, which is also in agreement with the observation made by Zhong et al., is that in our study, the processing with Sof-Lex abrasive discs was carried out on enamel surfaces that had not been previously subjected to dental stripping [55].

The scientific literature presents divergent views on the impact of enamel polishing. Initial investigations have indicated that grooves and abrasions resulting from interproximal reduction are resistant to removal through polishing techniques [4,21,22,23,47]. However, contemporary studies propose that subsequent to enamel stripping, polishing can render the enamel surface superiorly smooth compared to its untreated state [24,55,56]. This advancement might be attributable to recent innovations in polishing materials. Current consensus within the research community strongly advocates for comprehensive polishing following interproximal reduction [23,24,47,55,56].

The study conducted by us thus indicates that grinding and the use of abrasives with coarse granularity (40; 60; 90 μm) ensure an efficient grinding process with increased yield in terms of removing irregularities from the enamel surface, but leave behind increased roughness unsuitable for tooth health. These poor physical characteristics can facilitate the deposition of bacterial plaque and favor acid erosion, leading to the occurrence of dental caries. Therefore, the use of 15 μm abrasive strips for surface uniformity and polishing with fine discs is imperative, in this way obtaining a surface similar to that of healthy enamel.

Following interdental stripping, it is essential to provide instructions on hygiene practices, encompassing interproximal plaque management and preventive actions like the application of topical fluoride to prevent adverse outcomes associated with the procedure.

Thus, from all the presented data, we can affirm that the null hypothesis initially proposed for this study has been rejected. The various methods of interproximal enamel reduction negatively influenced the surface roughness of the tooth, particularly the 90 μm abrasive strips and diamond burs, where we obtained statistically significant differences compared to the values obtained in the control group. However, for optimal preservation of the surface microstructure and nanostructure, the clinical recommendation is to use abrasive discs for finishing and polishing after IPR, as they ensure the safe and effective completion of interproximal enamel reduction.

Potential limitations of this study may arise from the inherent dissimilarity between silicone and the periodontal ligament, as silicone is likely to experience fatigue more rapidly than a biological tissue. In an in vitro investigation, increasing the duration of stripping processes results in a higher number of force applications on both the tooth and the silicone. Applying stripping to the silicone bases may cause these teeth to become less secure compared to teeth that have not undergone this process. Loose teeth are unable to withstand the mechanical motion of the stripping equipment and, as a result, they are not effectively ground. 

Additional elements that can complicate the analysis include the potential for experimental inaccuracies and variations in the microstructure of the enamel between different samples. 

Furthermore, the surface roughness of enamel is strongly influenced by the mechanical and chemical processes that continuously occur in the oral cavity. It is very challenging to accurately replicate natural biological conditions, which underscores the need for more detailed subsequent studies that closely approximate the in situ conditions of the oral cavity.

Additionally, we did not differentiate tooth types into groups (incisors, canines, premolars, or molars) because the surface structure of the enamel on the mesial and distal surfaces is uniform at the 0.5 mm depth. However, future studies could explore any microstructural differences that might exist.

This study did not investigate any biological responses—namely, the reactions of the pulp–dentin complex during and after therapeutic stripping treatments. Future ex vivo and in vivo experiments will focus on studying the impact of force/pressure applied to the tooth during IPR and the temperature created by various stripping tools. Moreover, our objective is to investigate the roughness of the enamel surface following dental stripping, specifically focusing on the effects of remineralization products often used in dental treatment. This will be explored in future studies.

## 5. Conclusions

In this in vitro investigation, SEM and AFM imaging analyses have revealed that various instruments used for IPR produce enamel surfaces with varying nanotopographies and degrees of roughness. The surfaces that underwent treatment with diamond burs had the highest level of roughness, whereas those treated with 90 μm and 60 μm abrasive strips followed with progressively lower levels of roughness. The 40 μm polisher had a milder effect on the dental enamel, while 15 μm abrasive strips and polishing discs ensured good preservation of the enamel surface quality and also facilitated the removal of natural wear traces present in the standard sample. Thus, surfaces treated with Sof-Lex discs and 15 μm abrasive strips generated the lowest surface roughness.

## Figures and Tables

**Table 1:** Artificial saliva components.

Composition	Percentage %
Na_2_HPO_4_ NaHCO_3_ CaCl_2_	0.3
HCl-1M	0.3
H_2_O	99.4

**Table 2:** IPR instruments used in the study.

IPR Instrument	Model	Manufacturer	Grit	Handpiece
Abrasive strips 15 microns	EVA active on both sides	Task Inc., Tokyo, Japan	15 μm	Slow speed (5000 rpm)
Abrasive strips 40 microns	EVA active on both sides	Task Inc., Tokyo, Japan	40 μm	Slow speed (5000 rpm)
Abrasive strips 60 microns	EVA active on both sides	Task Inc., Tokyo, Japan	60 μm	Slow speed (5000 rpm)
Abrasive strips 90 microns	EVA active on both sides	Task Inc., Tokyo, Japan	90 μm	Slow speed (5000 rpm)
Burs	8392 “mosquito” bur	Komet, Rock Hill, SC, USA	Red Fine grit	High speed (400,000 rpm) with water cooling
Discs	Sof Lex System Kit	3M, Neuss, Germany	Orange Fine 20 μm	Slow speed (5000 rpm)

**Table 3:** Multiple comparisons of roughness (Peak Height parameter) created by IPR instruments; Kruskal–Wallis Test.

Peak Height (nm)	Control	15 μm Abrasive Strips	40 μm Abrasive Strips	60 μm Abrasive Strips	90 μm Abrasive Strips	Diamond Burs	Abrasive Discs	*p*-Value
Number of values	15	15	15	15	15	15	15	
Minimum	51.00	48.00	21.00	79.00	110.0	115.0	33.00	
Median	153.5	147.0	216.0	179.5	415.0	309.0	123.0	0.0120 *
Maximum	594.0	661.0	691.0	958.0	1600	2800	1800	
Mean	231.1	232.5	254.1	289.6	602.2	540.0	284.5	
Std. Deviation	185.8	206.9	204.8	249.1	501.5	681.0	455.0	

* Indicates significant *p* value (*p* < 0.05).

**Table 4:** Multiple comparisons of roughness (Ra parameter) created by IPR instruments; Kruskal–Wallis Test.

Ra (nm)	Control	15 μm Abrasive Strips	40 μm Abrasive Strips	60 μm Abrasive Strips	90 μm Abrasive Strips	Diamond Burs	Abrasive Discs	*p*-Value
Number of values	15	15	15	15	15	15	15	
Minimum	5.610	5.970	2.490	10.20	7.940	16.20	4.610	
Median	14.15	16.45	26.70	23.05	54.10	31.75	12.45	0.0042 *
Maximum	60.90	80.40	73.10	138.0	159.0	217.0	155.0	
Mean	22.01	25.57	26.29	34.34	64.04	50.74	28.58	
Std. Deviation	17.49	22.60	20.31	33.02	46.15	51.75	39.60	

* Indicates significant *p* value (*p* < 0.05).

**Table 5:** Multiple comparisons of roughness (Rq parameter) created by IPR instruments; Kruskal–Wallis Test.

Rq (nm)	Control	15 μm Abrasive Strips	40 μm Abrasive Strips	60 μm Abrasive Strips	90 μm Abrasive Strips	Diamond Burs	Abrasive Discs	*p*-Value
Number of values	15	15	15	15	15	15	15	
Minimum	2.600	7.450	3.140	12.60	10.50	20.30	5.730	
Median	20.45	20.70	36.90	28.45	63.45	42.70	15.40	0.0045 *
Maximum	74.90	99.80	88.50	162.0	203.0	282.0	208.0	
Mean	28.11	32.55	34.29	43.09	81.69	65.80	37.04	
Std. Deviation	23.37	28.82	26.00	39.03	59.82	67.01	52.93	

* Indicates significant *p* value (*p* < 0.05).

## Data Availability

Data are contained within the article.
